# Artificial Synapses Based on Bovine Milk Biopolymer Electric-Double-Layer Transistors

**DOI:** 10.3390/polym14071372

**Published:** 2022-03-28

**Authors:** Sung-Hun Kim, Won-Ju Cho

**Affiliations:** Department of Electronic Materials Engineering, Kwangwoon University, Gwangun-ro 20, Nowon-gu, Seoul 139-701, Korea; tjdgns0721@naver.com

**Keywords:** artificial synapse, biocompatible, bovine milk, casein, lactose, proton, synaptic transistor, electric double layer

## Abstract

With the growing demand for bio- and eco-friendly artificial synapses, we propose a novel synaptic transistor using natural bovine-milk-based biocompatible polymers as an electrical double layer (EDL). A method for forming an EDL membrane, which plays a key role in synaptic devices, was established using a milk-based biocompatible polymer. The frequency-dependent capacitance of a milk-based polymer-EDL was evaluated by constructing an EDL capacitor (EDLC) with indium-tin-oxide (ITO) electrode. As a result, a significantly large capacitance (1.48 μF/cm^2^ at 1 Hz) was identified as an EDL effect due to the proton charge of the bovine-milk-based polymer, which is much more superior compared to conventional insulating materials such as SiO_2_. Subsequently, by using a milk-based polymer-EDL membrane in the fabrication of electronic synaptic transistors, we successfully implemented important synaptic functions, such as paired-pulse facilitation, dynamic filtering, and synaptic-weight-integration-based logic operations. Therefore, the proposed milk-based biocompatible polymer-EDL membrane offers new opportunities for building eco-friendly and biodegradable artificial synaptic systems.

## 1. Introduction

Recently, bio-inspired devices have attracted considerable attention because of their ability to implement and explore the superior functions of biological systems. In particular, artificial synapses are of great significance for neuromorphic systems because they can mimic the signal processing and memory functions of biological synapses and neurons [1,2,3]. However, traditional silicon-based integrated circuits (ICs) have limitations in that they consume considerably more energy than biological synapses. Therefore, it is imperative to develop scalable and low-power devices for neural systems at the human brain level [4,5,6]. These demands have been met with polymer-electrolyte-based synaptic transistors using a polymer electrolyte material as an insulating layer. The main advantage of polymer-electrolyte-based transistors is their low driving voltages. Compared to conventional insulating materials (e.g., 300 nm thick SiO_2_ is~10 nF/cm^2^), the electrolyte has a very large capacitance (~1 μF/cm^2^) due to the electric double layer (EDL) effect; therefore, the driving voltage and energy consumption of the transistor can be drastically reduced [7,8]. Recent advances in applying natural biomaterials to construct functional electronic devices have received increased attention for their use in environment-friendly and biocompatible electronic systems. Protein is a biomaterial that is a rich resource and essential natural component of all organisms. In the past few years, protein-based materials such as egg albumin, fish protein collagen, silk fibroin, and keratin have been applied to biocompatible artificial neuromorphic devices [9,10,11]. Meanwhile, bovine milk is a structurally excellent and useful protein that has been used as a material for making plastics since 1897 [12,13,14]. Thankfully, it is a rich bio-friendly protein that is easily obtainable from bovine cows.

In this study, we propose a novel type of artificial synapse based on a natural bovine milk polymer-EDL. The EDL effect was demonstrated by evaluating the frequency-dependent capacitance properties based on proton charges in the bovine milk component. We fabricated synaptic transistors by applying an indium-tin-oxide (ITO) bottom gate to the pre-synapse, a bovine milk polymer-EDL to the neurotransmitter, and an indium-gallium-zinc oxide (IGZO) channel to the post-synapse. Subsequently, synaptic behaviors were successfully implemented through slow polarization of mobile protons in the bovine milk polymer-EDL. In addition, important synaptic functions such as changes in the hysteresis voltage over the DC gate voltage sweep range, the excitatory postsynaptic current (EPSC) for AC gate spike stimulation, and logic operations have been demonstrated.

## 2. Experimental Section

### 2.1. Fabrication of Bovine Milk Polymer Electric-Double-Layer Transistors

**Figure 1:**
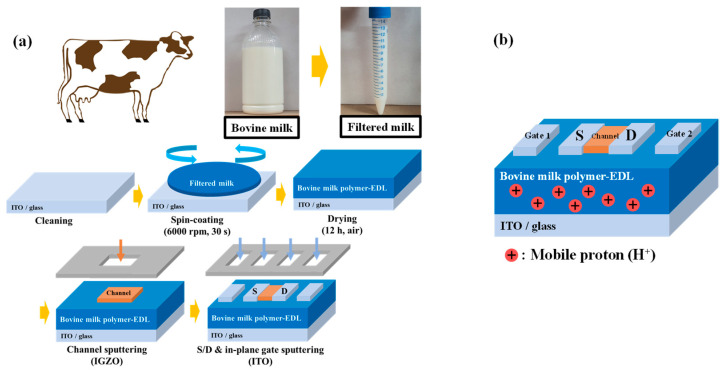
(**a**) Fabrication process and (**b**) structure of bovine-milk-polymer-based synaptic transistors.

The bovine-milk-polymer-based synaptic transistor was fabricated on a cleaned Corning 7509 glass substrate (Corning Inc., New York, NY, USA). For the bottom gate electrode, a 300-nm-thick ITO film (ITO target, THIFINE, Incheon, Korea) was deposited using an RF magnetron sputtering at an RF power of 100 W, a working pressure of 3.0 mTorr, and an Ar gas flow rate of 20 sccm. The bovine milk polymer-EDL membrane was prepared from commercial milk (Seoul Milk, Seoul, Korea). The milk was filtered through a 1-μm-pore-size polytetrafluoroethylene (PTFE) syringe filter (Whatman, Maidstone, UK) to remove the particulates. The filtered milk solution was then spin-coated at 3000 rpm and dried in air for 12 h under a relative humidity and temperature maintained at 20% and 25 °C, respectively. The thickness of the solid bovine milk polymer-EDL membrane was 200 nm (±10 nm deviation). Then, 50-nm-thick IGZO channels (IGZO target ratio: In_2_O_3_/Ga_2_O_3_/ZnO = 4:2:4.1 mol.%, THIFINE) were deposited using an RF magnetron sputtering at an RF power of 100 W, a working pressure of 6.0 mTorr, and an Ar gas flow rate of 30 sccm with a shadow mask. The channel width (W) and length (L) of the fabricated synaptic transistors were 1000 and 80 μm, respectively. Finally, 150-nm-thick ITO (ITO target, THIFINE) source/drain and in-plane gate electrodes (1000 μm × 200 μm) were formed using RF magnetron sputtering and a shadow mask. The distance between the source/drain and the in-plane gate electrodes was 300 μm. Figure 1 shows the fabrication process flow and the structure of the bovine-milk-polymer-based synaptic transistor.

### 2.2. Characterization

The surface morphologies and thicknesses of the bovine milk-EDL membranes were examined using a DektakXT Bruker stylus profiler (Bruker, Hamburg, Germany). The optical transmittances of the bovine milk-EDL membranes were measured using an Agilent UV–vis spectrophotometer (Agilent Technologies, Santa Clara, CA, USA) in the wavelength range of 400–1000 nm. The frequency-dependent specific capacitance of the ITO/bovine-milk-EDL/ITO-structured capacitor was analyzed using a 4284A Precision LCR meter (Hewlett-Packard Co., Palo Alto, CA, USA). The electrical characteristics of the fabricated bovine-milk-based synaptic transistor were evaluated using an Agilent 4156 B precision semiconductor parameter analyzer (Hewlett-Packard Co.). Electrical presynaptic pulse stimulation was applied using an Agilent 8110A pulse generator (Hewlett-Packard Co.) to measure synaptic behavior. The synaptic transistors were placed in a shielded dark box during the electrical measurements to prevent any external electrical and optical noise.

## 3. Results and Discussion

### 3.1. Property Verification of Bovine Milk Polymer Electric-Double-Layer Membrane

**Figure 2:**
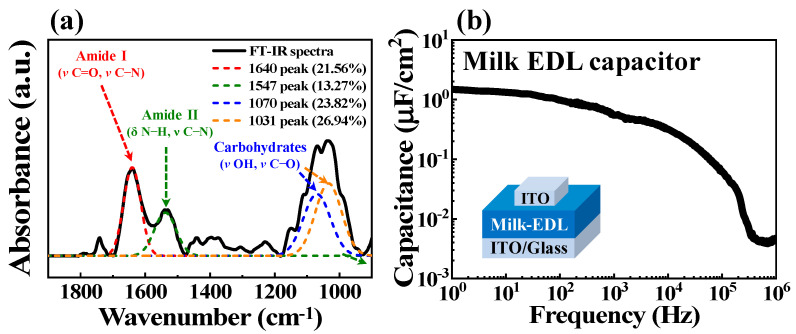
(**a**) FT-IR spectrum of the dried bovine milk-EDL membrane. (**b**) Frequency-dependent capacitance of ITO/milk-EDL/ITO-structured capacitors.

Several properties of the novel bovine-milk-based EDL material must be verified before using them in synaptic transistors. Bovine milk is composed of approximately 88% water, 3.2% fat, 3.2% protein, 4.5% lactose, and 0.7% minerals. To confirm the composition of spin-coated and sufficiently dried bovine milk-EDL membranes in air, Fourier transform infrared spectroscopy (FT-IR) was performed; the FT-IR spectra are shown in Figure 2a. The most relevant FT-IR peaks of bovine milk are in the 1900–900 cm^−1^ wavelength range. The range of 1700–1200 cm^−1^ is a mixed region with vibrational bands of fatty acids, proteins, and polysaccharides. The range of 1200–900 cm^−1^ is a region dominated by polysaccharide peaks. Among these, four main peaks were observed: the 1640 cm^−1^ peak represented amide I (ν C=O, ν C–N), the peak at approximately 1547 cm^−1^ represented amide II (δ N–H, ν C–N), and the peaks at approximately 1070 cm^−1^ and 1031 cm^−1^ represented carbohydrate (such as lactose) peaks (ν OH, ν C–O) [15,16,17,18,19]. For a detailed quantitative analysis of these main peaks, deconvolution was performed, and the areas were represented as percentages considering the total area under the parent peak [20,21]. In the 1900–900 cm^−1^ wavelength range, the amide I and II peaks were 21.56% and 13.27%, and the carbohydrate peaks were 23.82% and 26.94%, respectively. Therefore, protein- and lactose-based peaks, commonly observed in bovine milk FTIR spectra, were predominantly observed; approximately 80% of these proteins are casein proteins. These representative components, casein, and lactose are abundant in mobile protons [22,23,24,25]. Due to the presence of these protons in milk, the EDL effect may occur at the interface when the milk-EDL membrane is formed. A high-density proton charge accumulates through the EDL effect, allowing it to respond to synaptic spikes through a strong capacitive coupling effect [26]. An EDL capacitor with an ITO/milk-EDL/ITO structure was fabricated to verify the EDL effect, and its frequency-dependent capacitance characteristics were measured. As seen in Figure 2b, the capacitance increases as the frequency decreases, and a high capacitance of 1.48 μF/cm^2^ was observed at 1 Hz. This high capacitance difference occurs because of the insufficient response time for protons to migrate to the interface at high frequencies, whereas large interfacial EDL capacitances are formed at low frequencies [9]. The large capacitance at low frequencies is due to the formation of EDL. When an electric field is applied, mobile protons move to a thin boundary layer at the interfaces, which results in the formation of the EDL. Therefore, a large capacitance was obtained due to the interfacial EDL effect. The frequency-dependent capacitance of EDL is related to ionic polarization [27]. Therefore, it was verified that the EDL effect appeared in the novel bovine-milk-based EDL membrane.

**Figure 3:**
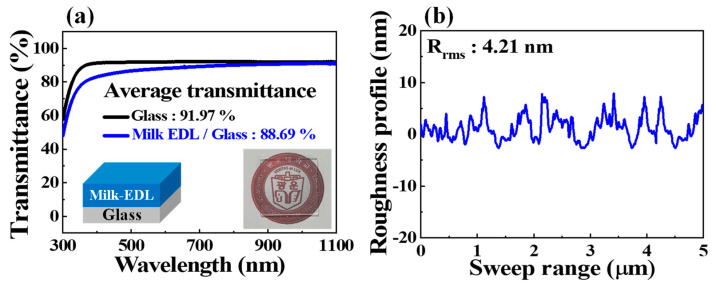
(**a**) Optical transmittance of bovine milk polymer-EDL membrane on a glass substrate. (**b**) Surface roughness properties of bovine milk polymer-EDL membrane.

The optical and surface properties were examined to verify the fabrication process’s suitability for spin-coated biocompatible bovine milk-EDL membranes. The transmittance of the milk-EDL membrane coated on a glass substrate is shown in Figure 3a. For comparison, the condition of the milk-EDL membrane (200 nm thickness) coated on the glass substrate (700 nm thickness) and the condition of the glass substrate by itself were measured, respectively. The average transmittance was extracted in the visible light range (400–1100 nm) through the transmittance characteristics: 91.97% for the glass-only substrate and 88.69% for the milk-EDL/glass substrate. As confirmed by the inset photo image, the milk-EDL/glass substrate showed an excellent transmittance that was approximately 3% lower than that of the glass substrate. Such excellent transmittance can be used in various applications, such as wearable and skin-attachable devices, to which biocompatible devices can be attached. Figure 3b shows the surface roughness of the bovine milk-EDL membrane in the 5 μm range, measured using a DektakXT Bruker stylus profiler. The root-mean-square (R_rms_) of the milk-EDL membrane calculated from the roughness characteristics was 4.21 nm. The results indicate that the bovine milk-EDL membrane surface has acceptable smoothness for reliable fabrication. In addition, in the micro fabrication process, the thinner the thickness of the EDL film applied as a dielectric, the more advantageous it is in terms of miniaturization. As can be seen in Table 1, it can be confirmed that EDL properties are well expressed despite the relatively thin EDL thickness compared to other previously reported protein-based EDL materials (applied to synaptic transistors).

### 3.2. Electrical and Neuromorphic Properties of Bovine Milk Polymer Electric-Double-Layer Transistor

**Figure 4:**
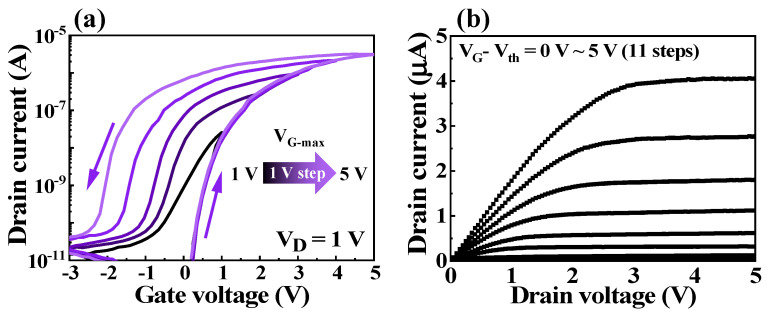
(**a**) Double-sweep transfer (I_D_−V_G_) curves according to maximum V_G_ (1 to 5 V, 1 V increments). (**b**) Output curves (I_D_−V_D_) according to V_G_−V_th_ (0 to 5 V, 0.5 V increments).

Since we verified the EDL effect of the bovine milk membrane by internal mobile protons, we applied the bovine milk-EDL to a synaptic transistor and measured its electrical properties. Figure 4a shows the double-sweep transfer (I_D_-V_G_) curves of the bovine-milk-EDL-based synaptic transistors. The transfer curves were continuously measured by increasing the maximum gate bias (V_G-max_) sweep range from 1 to 5 V (in 1 V increments) at a constant drain voltage (V_D_) of 1 V. Hysteresis in the counter-clockwise direction appeared in the double-sweep transfer curves and widened as V_G-max_ increased, indicating a slow polarization reaction for the mobile protons in the bovine milk-EDL. The more the protons accumulate at the channel interface due to the stronger positive gate bias, the stronger the negative gate bias required to diffuse them back, and the wider the counter-clockwise hysteresis. Figure 4b shows the output (I_D_-V_D_) curves. The output curves were measured at V_G_–V_th_ values from 0 to 5 V in increments of 0.5 V. As V_D_ increased, I_D_ increased linearly and became saturated, indicating stable pinch-off characteristics.

**Figure 5:**
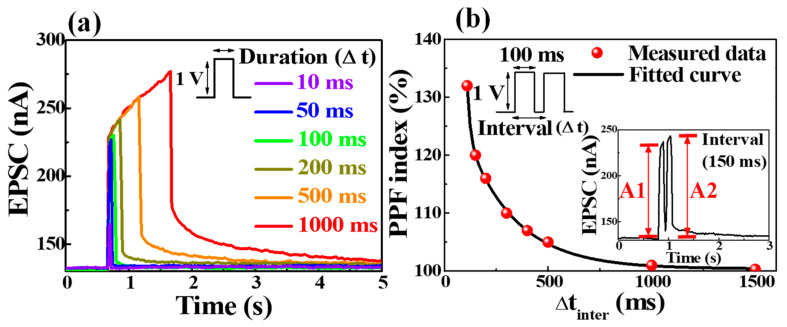
(**a**) EPSC retention characteristics of a single pre-synapse spike with duration (10 to 1000 ms) variation. (**b**) PPF index (A2/A1 × 100%) plotted as a function of spike interval times. The inset represents EPSC triggered by paired spikes at 100 ms intervals.

The synaptic behavior of the synaptic transistor operation is essential to mimic the biological synapse’s structure and mechanism. In biology, neurons and synapses behave like two basic computational engines in the brain. Signal spikes generated by presynaptic neurons are transmitted to postsynaptic neurons through neurotransmitters [30]. In the fabricated bovine-milk-EDL-based synaptic transistor, electrical spikes are generated at the ITO bottom gate (pre-synapse) and transmitted through the bovine milk-EDL (neurotransmitter) to the IGZO channel (post-synapse). Electrical spikes generated by the pre-synapse gate cause a mobile proton excitatory current in the post-synapse channel, called the excitatory postsynaptic current (EPSC); EPSC is a basic representation of synaptic strength [31]. Figure 5a shows the EPSC retention curves for the gate presynaptic spikes, with an amplitude of 1 V for spike duration variations (10–1000 ms). The longer the stimulus duration, the higher the concentration gradient due to the migration of mobile protons in the bovine milk-EDL, thus slightly increasing the EPSC retention time. A few seconds of EPSC retention time implies short-term synaptic plasticity, facilitating the learning and memory functions of the nervous information process system [32]. As a form of short-term synaptic plasticity, the paired-pulse facilitation (PPF) characteristic represents the dynamic enhancement of neurotransmitters in biological neural synapses and is involved in various neural tasks, such as simple learning and encoding temporal information [33]. The PPF index can be obtained as the ratio of the EPSC value triggered by the first spike (A1) to the EPSC value triggered by the second spike (A2). Figure 5b shows the paired-pulse facilitation (PPF) index, where two consecutive spikes with an amplitude of 1 V and a duration of 100 ms were applied by adjusting the interval (Δt_inter_) between the spikes. The shorter the interval between the two spikes, the more mobile protons accumulate before diffusing back, and the more EPSC facilitation occurs. At 100 ms intervals, the PPF index increased to 132%.

**Figure 6:**
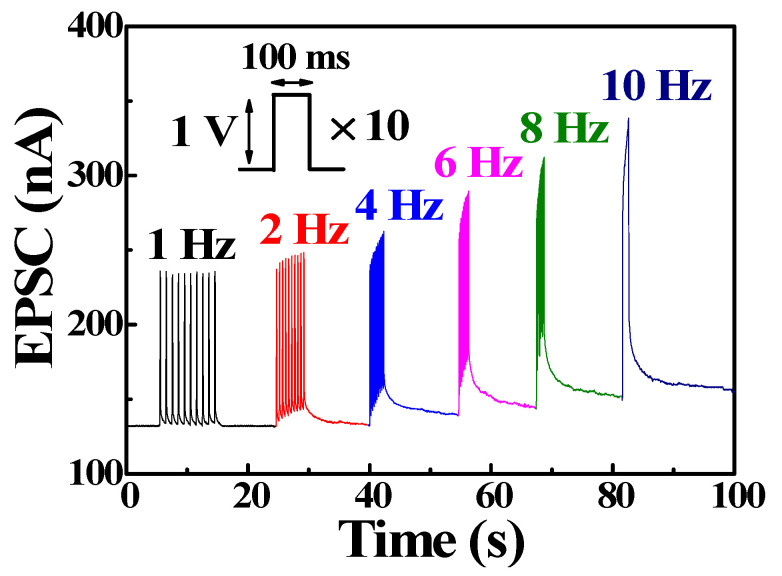
Dynamic filter behavior recorded in response to 10 cycles of spikes at different frequencies (1 to 10 Hz).

Based on the EPSC retention, the synaptic transistor can also act as a dynamic filter for spike information transmission, depending on the spike signal frequency. Short-term synapse depression contributes to low-pass temporal filtering, whereas short-term synapse facilitation contributes to high-pass temporal filtering [26,34]. Figure 6 shows the dynamic filter behavior of the EPSC responses to 10 spike cycles (amplitude 1 V, duration 100 ms) at different frequencies. At a frequency of 1 Hz, the peak EPSC value remained at ~230 nA, even after 10 spike cycles. As the frequency increased, the peak value of the EPSC increased, indicating that the bovine-milk-EDL-based synaptic transistor can act as a dynamic high-pass filter for information transmission.

**Figure 7:**
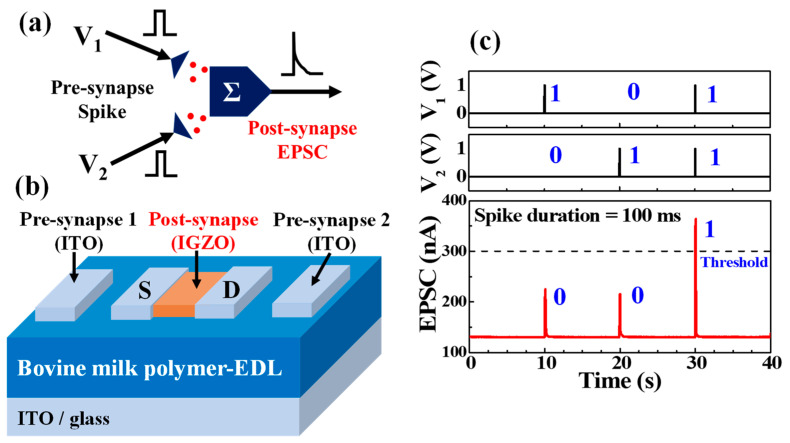
(**a**) Schematic diagram of synaptic weight integration. (**b**) Structure of two in-plane gated bovine-milk-based synaptic transistors for synaptic weight integration realization. (**c**) AND logic operation tuned by summation of two synchronous pre-synapse spikes.

Thus far, we have implemented a temporal synaptic plasticity function for a bovine-milk-EDL-based synaptic transistor. In actual neurons, thousands of synaptic inputs arrive at different dendritic locations. These synaptic weights are integrated into neurons and trigger local outputs, which is one of the basic principles of neuronal computation [35,36,37]. This synaptic weight integration enables the realization of the EPSC-based AND logic of the synaptic transistor. Figure 7a,b show a schematic of the synaptic weight integration caused by two different presynaptic spikes. Two-sided in-plane gates with a gate-coupling effect were applied as two presynaptic input terminals. Figure 7c shows the AND logic operation tuned by the summation of the two presynaptic spikes. The conditions for a single pre-synapse spike were a 100 ms duration and a 1 V amplitude. When only one presynaptic spike was applied, the EPSC peak value was ~230 nA. However, when two presynaptic spikes are simultaneously applied on two presynaptic inputs, the EPSC exceeds the threshold of ~370 nA arbitrarily set by the integrated synaptic weight, outputting a value of 1. Considering these results, various information configurations are possible using the synaptic weight integration property, thus providing the foundation for implementing multiple and complex synaptic spatial information summation in the human brain.

## 4. Conclusions

In this paper, we propose a novel artificial synaptic transistor using a natural bovine milk polymer-EDL. The EDL effect due to the abundant proton charge of the bovine milk membrane was confirmed through its frequency-dependent capacitance characteristics. In addition, various process applications of bovine milk-EDLs were secured through their excellent transmittance and surface properties. Thereafter, synaptic transistors were fabricated from bovine milk-EDLs with proven EDL effects. The double-sweep transfer curves of the bovine-milk-EDL-based synaptic transistor showed counter-clockwise hysteresis due to the slow polarization reaction and the back diffusion of mobile protons in the bovine milk-EDL. In addition, synaptic functions including paired-pulse facilitation, dynamic pass filtering, and synaptic weight integration-based AND logic operations were successfully implemented. Furthermore, if the EDL properties of each milk component are analyzed, its potential as a more precise biocompatible EDL material will be secured. The proposed bovine milk polymer-EDL is expected to provide new opportunities for building biocompatible artificial synapses and brain-inspired systems with protein-based polymer-EDL material.

## Figures and Tables

**Table 1:** Thickness benchmark of protein-based materials applied as EDL of synaptic transistors.

References	Protein-Based Material for EDL Dielectric	Spin-Coating Condition	Thickness
Ref. [9]	Egg-albumen	3000 rpm	1 μm
Ref. [28]	Gelatin	2000 rpm	664 nm
Ref. [29]	Keratin	2000 rpm	646 nm
This study	Milk	6000 rpm	200 nm

## Data Availability

The data presented in this study are available from the corresponding author upon reasonable request.
